# Suicide rates and income in São Paulo and Brazil: a temporal and spatial epidemiologic analysis from 1996 to 2008

**DOI:** 10.1186/1471-244X-12-127

**Published:** 2012-08-28

**Authors:** Daniel H Bando, Andre R Brunoni, Isabela M Benseñor, Paulo A Lotufo

**Affiliations:** 1 Doctoral Program of Sciences, Faculdade de Medicina, University of São Paulo, São Paulo, Brazil; 2 Clinical and Epidemiological Research Center, Hospital Universitário, University of São Paulo, São Paulo, Brazil; 3 Department of Neurosciences and Behavior, Instituto de Psicologia, University of São Paulo, São Paulo, Brazil; 4 Clinical and Epidemiological Research Center, Hospital Universitário, Av Lineu Prestes 2565, 3° andar – Centro de Pesquisas Clínicas, Cidade Universitária, São Paulo, SP, Brazil

## Abstract

**Background:**

In a classical study, Durkheim noted a direct relation between suicide rates and wealth in the XIX century France. Since that time, several studies have verified this relationship. It is known that suicide rates are associated with income, although the direction of this association varies worldwide. Brazil presents a heterogeneous distribution of income and suicide across its territory; however, evaluation for an association between these variables has shown mixed results. We aimed to evaluate the relationship between suicide rates and income in Brazil, State of São Paulo (SP), and City of SP, considering geographical area and temporal trends.

**Methods:**

Data were extracted from the National and State official statistics departments. Three socioeconomic areas were considered according to income, from the wealthiest (area 1) to the poorest (area 3). We also considered three regions: country-wide (27 Brazilian States and 558 Brazilian micro-regions), state-wide (645 counties of SP State), and city-wide (96 districts of SP city). Relative risks (RR) were calculated among areas 1, 2, and 3 for all regions, in a cross-sectional approach. Then, we used Joinpoint analysis to explore the temporal trends of suicide rates and SaTScan to investigate geographical clusters of high/low suicide rates across the territory.

**Results:**

Suicide rates in Brazil, the State of SP, and the city of SP were 6.2, 6.6, and 5.4 per 100,000, respectively. Taking suicide rates of the poorest area (3) as reference, the RR for the wealthiest area was 1.64, 0.88, and 1.65 for Brazil, State of SP, and city of SP, respectively (p for trend <0.05 for all analyses). Spatial cluster of high suicide rates were identified at Brazilian southern (RR = 2.37), state of SP western (RR = 1.32), and city of SP central (RR = 1.65) regions. A direct association between income and suicide were found for Brazil (OR = 2.59) and the city of SP (OR = 1.07), and an inverse association for the state of SP (OR = 0.49).

**Conclusions:**

Temporospatial analyses revealed higher suicide rates in wealthier areas in Brazil and the city of SP and in poorer areas in the State of SP. We further discuss the role of socioeconomic characteristics for explaining these discrepancies and the importance of our findings in public health policies. Similar studies in other Brazilian States and developing countries are warranted.

## Background

Suicide rates vary widely between and within countries, since it is a complex phenomenon, related to several singularities. Some general demographic risk factors are known as sex (men), age-strata (young, elderly) and ethnicity (European). Other factors contribute to suicide and include: genetic loading, personality characteristics (impulsivity, aggression), psychiatric and physical disorders (pain, incapacity), life events (loss, trauma), social isolation, availability of means, substance abuse, economic condition [1,2].

The highest suicide rates are in Eastern Europe (former USSR countries) and the lowest rates, in some Latin American countries [1,3]. Disparate geographic distribution of suicide has been recognized for the past two centuries, with the first seminal observations of Morselli [4] and Durkheim [5], who acknowledged an endemic pattern of suicide in the late XIX century in Europe. Morselli noted a higher suicide rate in Denmark and central Germany, while Durkheim observed the same in northern France, a finding confirmed recently by Baller & Richardson [6] in an analysis using the same data.

The first population-based suicide studies date from the early nineteenth century Europe, highlighted through the work of Jean-Pierre Falret, Esquirol disciple [7,8]. Later, many other studies emerged as led by Morselli, Masaryk, Guerry, Tarde, Winslow, Wagner [4,5,7,9]. Methodologically, the most consistent work belonged to Emile Durkheim, which combined the available empirical data with a well-defined sociological theory [5]. "The suicide”, Durkheim's masterpiece, inaugurated the modern sociology and was one of the first ecological studies, a major influence in epidemiology. Durkheim's theory is based on two concepts: social integration and social regulation. Suicidal behavior is common in societies where there is a low degree of social integration, culminating in the egoistic suicide. The individual is protected from egoism by religions with strong group ties (e.g. Catholic Church) and family ties. Suicidal behavior is also common in societies where there is a low degree of social regulation, culminating in the anomic suicide. Social regulation can be understood as external regulatory forces on the individual. Economic cycles (depression or prosperity) and income level are examples of factors that could modulate anomic suicide. Durkheim is commonly criticized for not providing a specific explicit definition of these social variables. Some derivations, extensions and reinterpretations of his theory were attempts to overcome such omission [10-13]. Despite the criticism, he remains one of the most well-known names of suicidology [14]. Sociologists, psychologists, epidemiologists, psychiatrists have used the same basic methodology established by Durkheim [3]. In the late XIX century, France history was marked by great economic development, which was accompanied by one of the highest suicide rates ever observed. When Durkheim claimed that "poverty protects against suicide," he based his observation on two area-based comparisons. He noted higher suicide rates in France compared to poorer countries, and also that suicide rates were higher in regions of France with greater wealth concentration. This finding raised the hypothesis that economic development could be related to individualism and, ergo, to social isolation and suicide. We aimed to evaluate the same relationship in Brazil and São Paulo regions using Geographic Information System (GIS) and statistical technics. However, the relationship between wealth and suicide is not straightforward; on the contrary, it is complex and changes throughout time and space. In Europe, for instance, different patterns were observed at the beginning of the twentieth century [15].

In this context, several methodological aspects hinder analysis of this phenomenon. For instance, different study designs have been used to evaluate suicide rates in relation to socioeconomic factors, although most have been ecological. There are also multiple indexes that can be used to evaluate socioeconomic characteristics (e.g. poverty/deprivation, unemployment, Gross Domestic Product (GDP), average income, etc.). Also, most studies were performed in developed countries. Partially because of these questions, results have been heterogeneous.

A systematic review of ecological studies in North America, Europe, Australia, and New Zealand, dating from 1897 to 2004, reported an inverse relation between socioeconomic characteristics and suicide [16]. Other recent ecological studies performed in the United States [17], Japan [18], Taiwan [19], Australia [20], England [21], Finland [22,23] and Italy [24] also demonstrated an inverse relationship. Moreover, an ecological study that grouped data from the G7 countries in 2007 observed an inverse relation between income and male suicides [25]. However, other ecological studies found different results. One worldwide, cross-sectional study performed in the late 1990s included data from 52 countries and identified a direct association between per capita GDP and male suicide rates in all regions except former socialist economy countries, which had abnormally high suicide rates [3]. Another study using World Health Organization (WHO) data focused on countries with a medium Human Development Index (HDI) and found that education and telephone density was directly related to suicide while a high Gini index was inversely related to suicide [26].

Longitudinal studies also present interesting findings. One study using data from 1960 to 1999 included data from 21 countries grouped into three categories according to GDP per capita; results showed notably that countries with higher income presented higher suicide rates than poorer countries [27]. Other longitudinal studies also observed this trend [28-30].

Therefore, the relationship between income and suicide varies, although it seems that it is mediated by socioeconomic development and income level. In this context, Brazil covers a wide area with almost 200 million inhabitants with very distinct cultural and socioeconomic characteristics, as shown by indexes such as the Gini coefficient and the HDI. In addition, although the overall suicide rate in Brazil is low, in Brazilian urban centers the rates vary from 5 to 15 per 100,000 [31-33]. Few studies have evaluated temporal trends of suicide in Brazil [34,35] and none has explored its relationship with income including spatial distribution.

Considering that (I) suicide and income are related, (II) Brazil presents important socioeconomic disadvantage, and (III) suicide rates in Brazil vary among location, the purpose of our study was to evaluate the relationship between suicide and income in Brazil and its regions. We also verified whether such relationship is related to socioeconomic characteristics.

## Methods

### Study design

**Figure 1:**
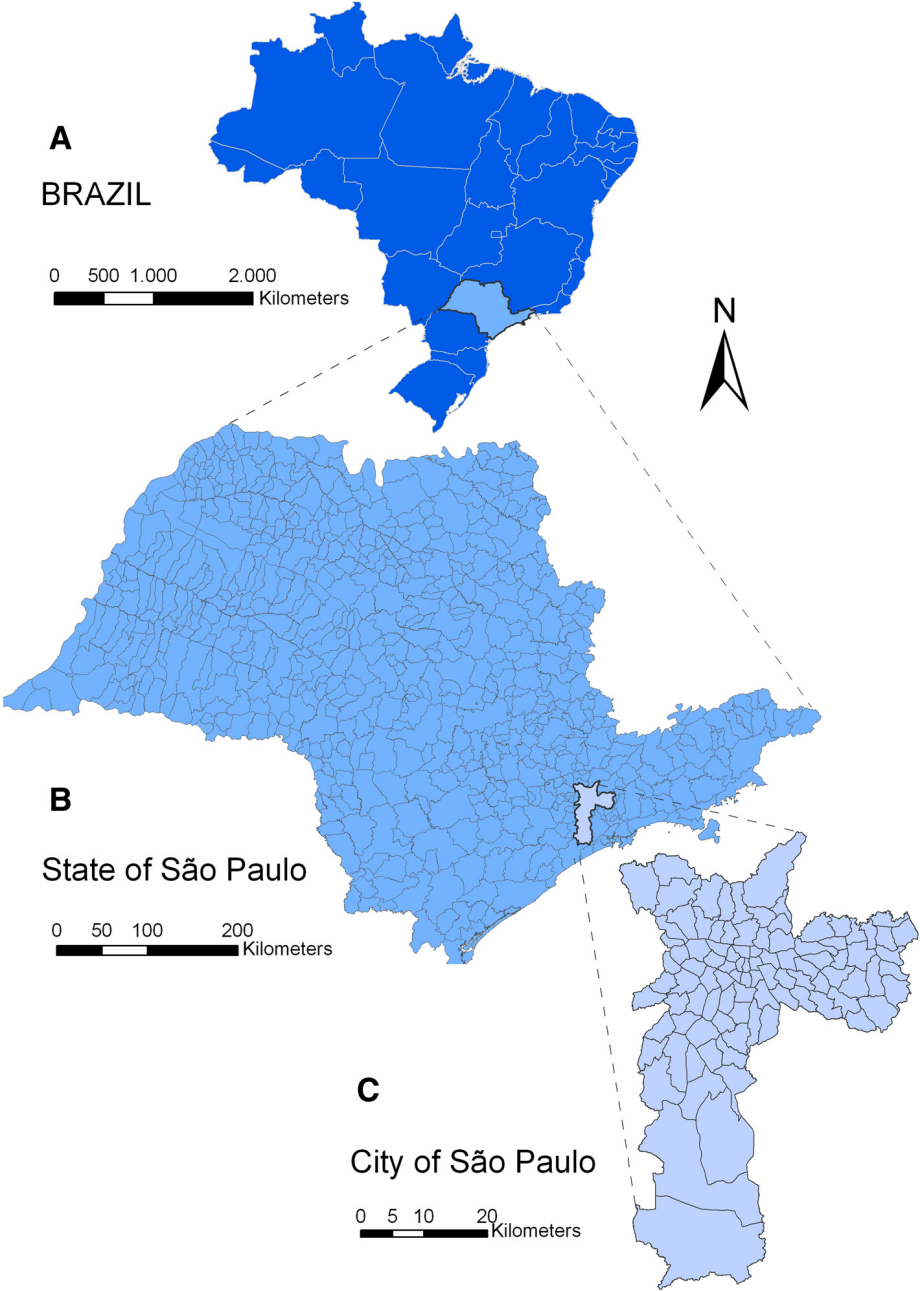
Study regions: A) Brazil, B) State of São Paulo, C) City of São Paulo.

We designed an epidemiological study considering suicide mortality data in three different regions (Figure 1): (1) a nation-wide comparison of the 27 States in Brazil and also its 558 micro-regions; (2) a within-state comparison, including the 645 municipalities in the State of São Paulo (SP); (3) a city-wide comparison, among the 96 neighborhoods that compose the city of São Paulo.

To explore our hypothesis that suicide, income, and socioeconomic disadvantage are related, we chose regions with distinct socioeconomic patterns. Regions were characterized by the Gini index, the HDI, and the GDP per capita. The Gini coefficient is a measure of income inequality, ranging from 0 (total equality) to 1 (total inequality). The HDI is based on life expectancy, education, and GDP per capita, also ranging from 0 (minimum development) to 1 (maximum development). Remarkably, these regions present distinct indexes of Gini, HDI, and GDP per capita (Table 1). We chose the city and State of São Paulo for analysis because considering economy and population aspects, they are the most relevant regions of Brazil.

**Table 1:** Sociodemographic aspects of the three study regions

**Study regions (composition)**	**Area (Km**^**2**^**)**	**Population (million)**	**Population density**	**HDI**	**Gini index**	**GDP per capita**
**Brazil**	8514876	170	20	0.766	0.606	6473
(27 States)						
**State of São Paulo**	248209	37	149	0.820	0.566	11345
(645 counties)						
**City of São Paulo**	1509	10	6915	0.841	0.620	15286
(96 neighborhoods)						

Population Density (inhabitants/Km^2^); HDI: Human Development Index; GDP: Gross Domestic Product (Brazillian Real – R$).

National Census (IBGE, 2000 [41]).

### Data extraction

We collected data from Brazilian and São Paulo State Death registry databases for the period 1996 to 2008. All the analyses were conducted on people aged 15 years and over separately for men and women [36]. The age-adjusted rates for suicide were calculated using the WHO standard population as a reference [37]. Rates are per 100,000 individuals per year. Deaths considered suicide were those that used codes corresponding to “intentional selfharm”, codes X60 to X84 according to the International Classification of Diseases and Deaths (ICD-10). We chose the data from 1996 onwards as to avoid bias, since during this period the ICD-10 was implemented. The databases assessed were those of the Ministry of Health National Unified System Department of Information Technology [38]. For the city of São Paulo, these data are reviewed by the Death Records Improvement Program, but is the same base [39]. All data are from official health statistics sources used in Brazilian ecological studies.

The total number of inhabitants in São Paulo and Brazil and average income per capita were based on the National Census (2000). Population projections was based on SEADE Foundation [40]. Average income is strongly correlated with Human Development Index (HDI), Gini index and education (For nation-wide approach the Pearson Correlation was: +0.92, -0.66, +0.87, respectively; p-value < 0.01). So, we decided to use the average income, because it is one of the most common socioeconomic indicators used in this study design [16]. It is a simple measurement that allows comparisons with other studies [26] and is a reliable information extracted from the National Census. All data of the investigated variables are of free access [38-41].

### Data synthesis

For each region, income was categorized in tertiles according to its distribution in three areas, from the wealthiest (area 1) to the poorest (area 3) [27].

### Data analysis

We performed the following analyses:

a) *Descriptive analysis,* cross-sectional approach (1996–2008), in which we described age-adjusted suicide rates per gender and income area in the three regions: Brazil, State of São Paulo, and city of São Paulo; relative risks (RRs) and 95% Confidence Interval (CI) were estimated using the poorest area as the reference.

b) *Temporal analysis,* in which we used Joinpoint Regression Program 3.4.3 (developed by National Cancer Institute [42]) to identify and estimate points of inflection in suicide rate trends. Joinpoint regression is a log-linear model that uses Poisson regression, creating a Monte Carlo permutation test to identify points where the trend line changes significantly in magnitude or in direction. The analysis starts with the minimum number of joinpoints (a zero joinpoint, which is a straight line) and tests whether one or more joinpoints are significant and should be added to the model (in our analysis, we allowed a maximum of three joinpoints). In the final model each joinpoint (if any was detected) indicates a significant change in the slope. The permutation test, which estimates the optimal number of joinpoints, was applied after all analyses at a significance level of 0.05. The annual percentage change (APC) and 95% CI were estimated for the time segments on both sides of the inflection points.

c) *Spatial analysis,* which employs a series of geographical information system (GIS) techniques and statistical methods. ArcGis was used to explore spatial patterns of suicide rates and average monthly income. SaTScan [43] was applied to identify the spatial clusters of high/low suicide rates across the territory. Spatial scan statistical test was implemented to detect whether the suicide cases were randomly distributed. In this analysis, the suicide RR of each spatial unit was calculated using a Poisson model, and the mean RR of each cluster (including one or more administrative unit) was also computed with the SaTScan. The average annual mortality of the whole period was defined as the reference for the RR in each cluster. The longitude and latitude of the centroids in each spatial unit were used in the analysis. The most likely clusters were indicated through the likelihood ratio test and indicated as circular windows, to test the hypothesis that these areas had a high/low rate as compared to other areas. Statistical significance of a given cluster was ascertained using Monte Carlo procedures. The final step consisted in verifying association between the suicide risk clusters and average income through logistic regression. The dependent logit variable contrasted risk cluster against the remaining non-risk cluster areas. Results were significant at a p level <0.05 and Hosmer and Lemeshow test was used to check model adjustment. Logistic regression analysis was carried out using Statistical Package of Social Sciences (SPSS), version 12.

## Results

### Descriptive analysis

Table 1 shows sociodemographic data by region. Income is more equally distributed in the State of São Paulo (according to the Gini Index) and less equally distributed in the city of SP. The city of SP presents the highest HDI and Brazil the lowest, although the city has also the highest GDP per capita (Table 1), which explains the high HDI index.

From 1996–2008, there were 98,904 suicides (78,723 men) in Brazil, 21,066 suicides (16,945 men) in the State of São Paulo, and 5,589 suicides (4,283 men) in the city of SP. The State of São Paulo presented the highest male/female ratio (4.4) and the city of São Paulo showed the lowest (3.7). Suicide rates in Brazil, the State of São Paulo, and the city of São Paulo were 6.2, 6.6, and 5.4 per 100,000 inhabitants, respectively (Table 2).

**Table 2:** Suicide rates by income and sex for the three regions, 1996–2008

**Regions**	**Sex**	**Rate per 100,000**			
		**Area 1 (wealthiest)**	**Area 2 (mid-income)**	**Area 3 (poorest)**	**All areas**
**Brazil**	Total	7.1	6.2	4.3	6.2
	Men	11.8	10.0	6.9	10.2
	Women	2.8	2.5	1.8	2.5
**State of São Paulo**	Total	6.6	6.9	7.4	6.6
	Men	11.0	11.3	12.3	11.1
	Women	2.5	2.4	2.5	2.5
**City of São Paulo**	Total	6.2	6.2	3.8	5.4
	Men	9.7	10.5	6.3	8.9
	Women	3.2	2.4	1.6	2.4

Taking the poorest area (area 3) as reference, the RR of suicide in more wealthy regions (areas 1 and 2) was significantly higher in Brazil and the city of São Paulo (Table 3). Conversely, the State of São Paulo showed an inverse relation, i.e. more wealthy areas were associated with a lower suicide risk. All RRs were significant, except for that of females in the State of São Paulo.

**Table 3:** Relative risk of suicide by income and sex for the three regions, 1996–2008

**Region**	**Sex**	**Relative Risk**		
		**Area 1 (wealthiest)**	**Area 2 (mid-income)**	**Area 3 (poorest)**
**Brazil**	Total	1.64* (1.63 to 1.68)	1.43* (1.40 to 1.46)	1
	Men	1.70* (1.66 to 1.73)	1.44* (1.40 to 1.47)	1
	Women	1.54* (1.48 to 1.59)	1.40* (1.34 to 1.46)	1
**State of São Paulo**	Total	0.88* (0.84 to 0.93)	0.92* (0.87 to 0.98)	1
	Men	0.89* (0.84 to 0.95)	0.92* (0.86 to 0.99)	1
	Women	1.03 (0.90 to 1.17)	0.98 (0.84 to 1.14)	1
**City of São Paulo**	Total	1.65* (1.54 to 1.77)	1.64* (1.53 to 1.76)	1
	Men	1.54* (1.42 to 1.67)	1.68* (1.55 to 1.81)	1
	Women	2.04* (1.76 to 2.36)	1.52* (1.31 to 1.77)	1

*statistically significant at p < 0.05.

### Temporal analysis

**Figure 2:**
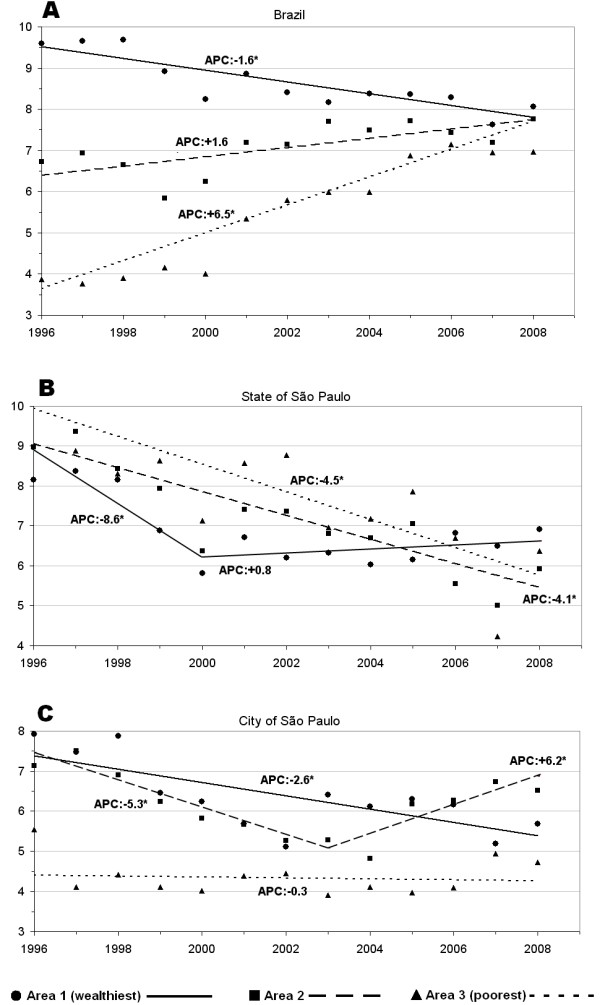
**Suicide trends by income areas in the three study regions.** *trend significantly different from zero APC: Annual Percentage Change Age-adjusted rates per 100,000.

All analyses considered data from 1996 to 2008. In Brazil, suicide rates in area 1 decreased significantly, while they increased in areas 2 and 3 (Figure 2A). In the State of SP, suicide rates in area 1 showed a significant decrease from 1996–2000, thereafter remaining stable until the end of the period. Rates in the other areas decreased significantly during the whole period (Figure 2B). Finally, in the city of SP, suicide rates in area 1 decreased significantly over the whole period; rates in area 2 decreased significantly up to 2003, and thereafter increased significantly until the end of the study period. Rates in area 3 remained stable (Figure 2C).

### Spatial analysis

**Figure 3:**
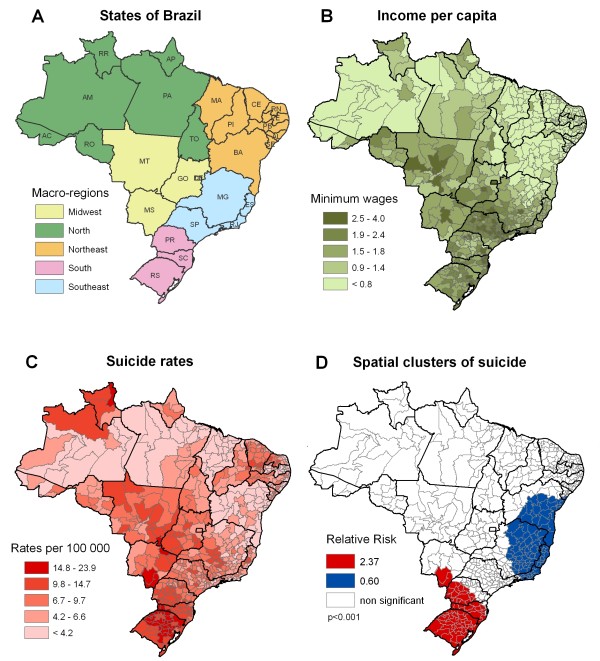
**(A) States of Brazil (AC: Acre, AL: Alagoas, AM: Amazonas, AP: Amapá, BA: Bahia, CE: Ceará, DF: Distrito Federal, ES: Espírito Santo, GO: Goiás, MA: Maranhão, MG: Minas, MS: Mato Grosso do Sul, MT: Mato Grosso, PA: Pará, PB: Paraíba, PE: Pernambuco, PI: Piauí, PR: Paraná, RJ: Rio de Janeiro, RN: Rio Grande do Norte, RO: Rondônia, RR: Roraima, RS: Rio Grande do Sul, SC: Santa Catarina, SE: Sergipe, SP: São Paulo, TO: Tocantins), (B) Income per capita, (C) Suicide rates, (D) Spatial clusters of suicide.** Source: IBGE (2000 [41]), DATASUS (2008 [38]).

The income map distribution for Brazil shows that the North and Northeast macro-regions had lower income than the Southeast and South regions (Figures 3A, 2B). The Southeast region presents the highest income per capita. On the suicide rate map (Figure 3C) rates were higher in the South, mostly in the State of Rio Grande do Sul. Central and Southeastern regions had intermediate rates, while North and Northeast regions had the lowest suicide rates.

The overall suicide rate in Brazil was 6.2 per 100,000. Spatial scan test identified two significant clusters: one cluster of high suicide rates (up to 12.7 suicides per 100,000), with a RR of 2.37, and another cluster of low rates (4.1 suicides per 100,000) with a RR = 0.60. The former cluster is located in the South region, encompassing 73 micro-regions; the latter cluster includes 98 micro-regions and comprises the east border, including the states of Rio de Janeiro, Espírito Santo, Minas Gerais and Bahia States (Figure 3). The cluster of high suicide rate showed a direct and significant association with income (Table 4).

**Table 4:** Association between suicide risk cluster and income, logistic regression

**Level**	**B**	**p**	**OR**	**95% CI**
Brazil	0.955	<0.01	2.598	1.840-3.669
State of São Paulo	−0.697	<0.01	0.498	0.370-0.671
City of São Paulo	0.067	<0.01	1.069	1.031-1.108

B: coefficient; p: significance; OR: Odds Ratio; CI95%: 95% Confidence Interval.

**Figure 4:**
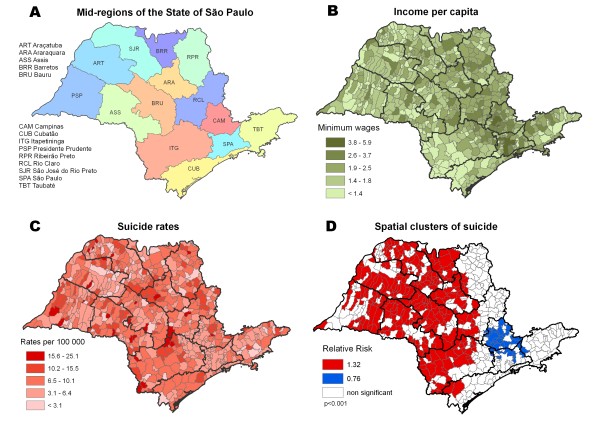
**(A) Mid-regions of the State of São Paulo, (B) Income per capita, (C) Suicide rates, (D) Spatial clusters of suicide.** Source: IBGE (2000 [41]), DATASUS (2008 [38]).

Income distribution in the state of SP showed that wealthier regions are concentrated in the mid-east, mostly around São Paulo and Campinas (Figures 4A, 4B), while poorer regions are in the south and west of the State. The overall suicide rate was 6.6 per 100,000 and the spatial pattern of suicide was unclear at visual inspection. The mid-region of São Paulo seemed to have lower rates, with outliers of higher rates to the west (Figure 4C).

Spatial scan test revealed a huge high-risk cluster with 8.2 suicides per 100,000 (RR = 1.32). A suicide protection cluster located in Campinas and north of São Paulo mid-regions was also identified (Figure 4D). Here an inverse and significant relation between suicide and income was observed (Table 4).

**Figure 5:**
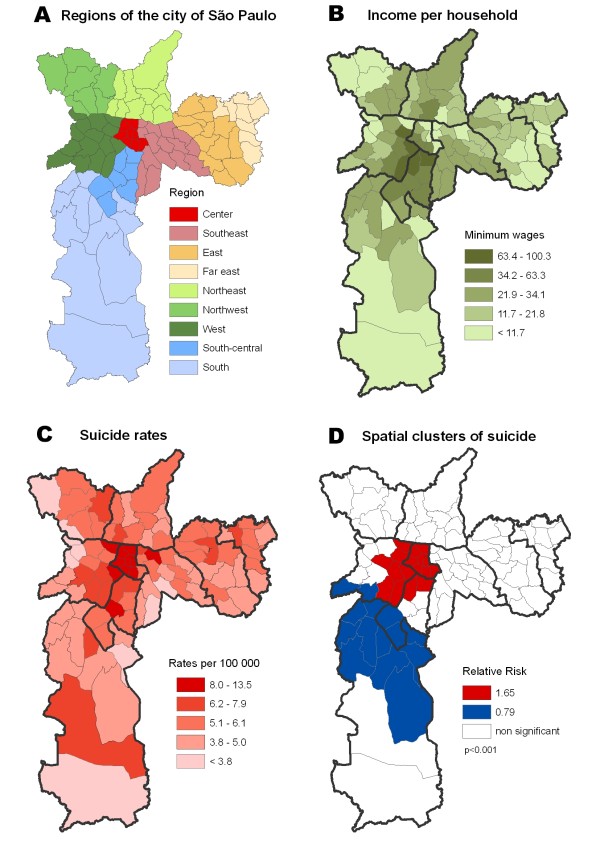
**(A) Regions of the City of São Paulo, (B) Income per capita, (C) Suicide rates, (D) Spatial clusters of suicide.** Source: IBGE (2000 [41]), DATASUS (2008 [38]).

Suicide rate in the city of SP was 5.4 per 100,000. The city has a high per capita income and high suicide rates in the central, south central and west areas (Figure 5A, 5B, 5C). Congruently, spatial analysis identified clusters of higher suicide risk in the wealthier areas (RR = 1.65). A cluster of suicide protection was also identified at the south region (Figure 5D). Thus, a direct and significant association between suicide risk and income was verified (Table 4).

## Discussion

Our study used ecological data to explore whether income and suicide were related in Brazil. Although ecological studies are considered methodologically problematic since they are based on aggregate data, ours has the strength of performing not only descriptive but also temporal and spatial analyses. While descriptive analysis assesses the magnitude of association between suicide and income in the different regions, the temporal analysis takes into account all these attributes over time. Finally, spatial analysis surveys for clusters of high suicide risk within regions. Taken together, these are useful tools for the evaluation and implementation of suicide interventions.

The descriptive approach showed that for Brazil and the city of SP, suicide rates were higher in wealthier areas; the opposite relation was found for the State of SP, i.e. the wealthiest area had the lowest suicide rate. Remarkably, this was confirmed by spatial analysis showing similar associations for all regions – i.e., a direct relation between income and suicide in Brazil and the city of SP, and an inverse relation for the State of SP.

Temporal analysis also showed interesting findings – one being that trends for different areas in Brazil and the State of SP converged at the end of the study period. This could be related to general improvements in health care and citizenship observed in Brazil in the last decade. On the other hand, the city of SP showed no such convergence. This could be related to the fact that the city maintained its high income inequality over the study period. Spatial analysis identified clusters with high and low suicide rates in Brazil, State of SP, and city of SP. Brazil had the highest relative risk of suicide, and also the lowest relative risk, comparing the south versus east/northeast regions. Considering the clusters of high suicide rates, the State of SP showed the lowest relative risk.

One important finding was that income and suicide directly related in Brazil and in the city of SP, but not in the State of SP, where suicide was inversely related with income. Interestingly, the State of SP presents a lower Gini index than the other regions. A systematic review of ecological studies from 1897 to 2004 showed that most studies (70%) reported an inverse association between income and suicide; most (88%) were also performed in developed countries [16]. Recent ecological studies conducted in developed countries show similar patterns, the majority finding an inverse relationship between suicide and income [17,18,20-23,25,44-46].

The same was noted in longitudinal approach studies [47-49] and using GIS techniques [19,50]. The spatial clusters of suicide in Japan were assessed using data from 1985 and 1995 for prefectures. Researchers used GIS techniques and models showed that suicide rates were higher in less populated areas, areas with more elderly persons, and in more religious areas. They suggested an influence of traditional Japanese culture that endorses suicide [51]. A study identified a marked change in the spatial epidemiology of suicide in young men from 2001 to 2005. Suicide rates in London had declined while they had increased in Wales [52]. In London neighborhoods, from 1996 to 1998, a study identified spatial dependency and a direct relationship between deprivation and suicide in males aged 30–49 years [53].

Some studies found a direct relation between suicide and income [3,26]. The lack of studies in developing countries still persists; in China (Shandong) an inverse relationship between suicide and income was identified [54]. Conversely, a study focusing on developing countries revealed a direct association between income (indexed by high education levels, high telephone density, and high cigarette consumption) and suicide. One possible explanation for these findings is that, in developing countries, social deprivation might be more accepted by the population, while in developed countries notions of self-worth and equality are valued [26]. Another interesting explanation is proposed by Paugam [55], after Castel [56]: authors who distinguish between *disqualifying* and *integrated* poverty. The latter, which best suits Brazil and the city of São Paulo (higher Gini coefficient), is observed in societies in which poverty is compensated by a solidarity response within family, neighborhood, and region. Integrated poverty was, in fact, the social reality that Durkheim referred when he claimed that “poverty protects” [3]. On the other hand, disqualifying poverty, which is more common in developed countries, might be related to demotion and loss of social status. Becoming poor in a rich society is, in fact, different than being poor in a poor society, and such loss of social status might lead to frustration and later to suicide [13].

The present study has some limitations. Different procedures and cultural and social practices and values probably have various effects on death records and lead to misclassification of suicide [1,2]. Another aspect is related with data quality. It is possible that the quality of the information have influenced suicide trends in Brazil. A study based on vital information provided by the Ministry of Health Brazilian system identified more deficient information in the poorest macro-regions [57], although this issue has improved in recent years. So, it is possible that the significant suicide increase in area 3 in Brazil could be an artifact. For the nation-wide comparison, we did a sensitivity analysis, based on two periods: 1996–2002 and 2003 onwards. We focused on area 3 because it was the poorest and presented the highest variation on suicide rates. We also analyzed the age-adjusted death rates by "Ill-defined and unknown causes of mortality" (code R99 of ICD-10). For the first period (1996–2002) suicide remained stable and death by “R99” showed a significant increase (APC = +12.2). For the second period (2003–2008) suicide increased significantly (APC = +3.7) and death by “R99” decreased significantly (APC = −15.2). There was an improvement in data quality from 2003 onwards, and also a slightly and significant increase on suicide rates. Thus, partially, we can assume that the significant suicide increase in area 3 in Brazil was due to an artifact, but suicide is a rare event, the APC was lower. Further research must be conducted to clarify these patterns. Spatial analysis using satscan has a limitation. For each area unit, the software considers the respective centroid and its radius, according to the suicide rate. This can bias the cluster detection, mostly in large areas with spatial variations in the population density. Despite this limitation in a recent review of software for space-time disease surveillance (include ClusterSeer, SaTScan, GeoSurveillance, Surveillance package for R), satscan was highlighted as the most developed and robust software for cluster detection [58].

## Conclusion

In conclusion, we performed a descriptive, temporal, and spatial analysis regarding income level and suicide in different regions of Brazil. Results showed that the direction of association varied, albeit resembling similar patterns observed worldwide, with a direct and inverse association in less and more unequal areas, respectively. Taken together, these findings suggest that income and suicide in Brazil are related, although socioeconomic regional characteristics might act as a moderator variable. Future studies comparing suicide rates in other Brazilian States and developing countries should take into account these results by performing separate analyses by region. Also, these results highlight that the association between suicide and income in Brazil varies importantly according to the considered region. The result of the present study is not conclusive. It helps to understand the phenomenon of suicide, generates new hypotheses for other study designs and can be useful for prevention strategies.

## Competing interests

The authors declare that they have no competing interests.

## Authors’ contributions

DHB undertook data extraction and analysis and wrote the first draft. ARB wrote the revised version. IMB and PAL formulated research questions and contributed to writing the drafts. All the authors contributed to the preparation of the final manuscript and approved the submission.

## Pre-publication history

The pre-publication history for this paper can be accessed here:

http://www.biomedcentral.com/1471-244X/12/127/prepub
